# Preclinical Profile and Characterization of the Hepatitis B Virus Core Protein Inhibitor ABI-H0731

**DOI:** 10.1128/AAC.01463-20

**Published:** 2020-10-20

**Authors:** Qi Huang, Dawei Cai, Ran Yan, Lichun Li, Yuhua Zong, Lida Guo, Alexandre Mercier, Yi Zhou, Ariel Tang, Kirk Henne, Richard Colonno

**Affiliations:** a Assembly Biosciences, South San Francisco, California, USA

**Keywords:** core protein, Cp, core inhibitor, cccDNA, hepatitis B virus, pregenomic RNA, antiviral agents, capsid, pgRNA packaging

## Abstract

ABI-H0731, a first-generation hepatitis B virus (HBV) core protein inhibitor, has demonstrated effective antiviral activity in chronic hepatitis B (CHB) patients in a phase 1b clinical trial and is currently being further evaluated in phase 2 clinical trials. Here, we report the preclinical profile of ABI-H0731. In *in vitro* cell culture systems (HepG2-derived cell lines HepAD38 and HepG2-NTCP and primary human hepatocytes [PHHs]), ABI-H0731 exhibited selective inhibition of HBV DNA replication (50% effective concentration [EC_50_] from 173 nM to 307 nM). Most importantly, ABI-H0731 suppressed covalently closed circular DNA (cccDNA) formation in two *de novo* infection models with EC_50_s from 1.84 μM to 7.3 μM. Mechanism-of-action studies indicated that ABI-H0731 is a direct-acting antiviral that targets HBV core protein, preventing HBV pregenomic RNA (pgRNA) encapsidation and subsequent DNA replication. The combination of ABI-H0731 with entecavir appears to decrease viral DNA faster and deeper than nucleoside/nucleotide analogue (NrtI) therapy alone. In addition, ABI-H0731 disrupts incoming nucleocapsids, causing the premature release of relaxed circular DNA (rcDNA) before delivery to the nucleus, and thus prevents new cccDNA formation. ABI-H0731 exhibits pangenotypic activity and is additive to moderately synergistic when combined with an NrtI. In addition to its potency and novel mechanism of action, ABI-H0731 possesses drug-like properties and a preclinical pharmacokinetic profile supportive of once-daily dosing in patients with CHB. Taken together, these data support the ongoing clinical development of ABI-H0731 as a treatment for HBV.

## Abstract (precis)

ABI-H0731, a first-generation hepatitis B virus (HBV) core protein inhibitor, has demonstrated effective antiviral activity in chronic hepatitis B (CHB) patients in a phase 1b clinical trial and is currently being further evaluated in phase 2 clinical trials. Here, we report the preclinical profile of ABI-H0731. In *in vitro* cell culture systems (HepG2-derived cell lines HepAD38 and HepG2-NTCP and primary human hepatocytes [PHHs]), ABI-H0731 exhibited selective inhibition of HBV DNA replication (50% effective concentration [EC_50_] from 173 nM to 307 nM).

## INTRODUCTION

Hepatitis B virus (HBV) infection is a major cause of chronic liver disease. The WHO estimates that in 2015, 257 million people had chronic HBV infection (CHB), with an estimated 887,000 deaths due to the end-stage complications of chronic HBV infection, cirrhosis and hepatocellular carcinoma (https://www.who.int/en/news-room/fact-sheets/detail/hepatitis-b). Current treatment options for CHB include nucleoside/nucleotide analogues (NrtIs) targeting the HBV polymerase-reverse transcriptase (HBV Pol-RT), and alpha interferon products. These therapies are successful in suppressing HBV replication in an appreciable proportion of CHB patients but are associated with low rates of clinical cures, requiring lengthy, if not lifelong, treatment to maintain viral suppression (1, 2). New therapies are required to provide patients with enhanced rates of clinical cure.

HBV is an enveloped virus belonging to the *Hepadnaviridae* family. Upon infection, its 3.2-kb, partially double-stranded DNA genome is delivered to the nucleus and repaired by host proteins to form a covalently closed circular DNA (cccDNA) molecule that harbors four overlapping open reading frames encoding the viral e antigen (HBeAg)/core protein (Cp), surface antigens (HBsAg), polymerase, and X protein (3). HBV Cp is a multifunctional protein at the center of the virus life cycle. Cp drives the assembly of icosahedral capsids and is directly involved in the recruitment and packaging of the viral pregenomic RNA (pgRNA), a biological process critical to HBV DNA replication and virus generation. Cp-mediated functions are also fundamental to HBV persistence since they directly impact the intracellular replenishment of nuclear pools of HBV cccDNA (4). Because Cp plays such a pivotal role in the HBV life cycle and possesses limited sequence polymorphism across HBV genotypes (5), Cp is now considered an attractive target for the design of direct-acting HBV antivirals. Several small molecules known as core protein inhibitors (CIs) have been reported to associate with Cp and interfere with the capsid assembly process (6–8). Indeed, CIs have been shown to block pgRNA encapsidation and prevent HBV DNA replication, which in turn negatively impact the production of infectious virions. Recent studies have also demonstrated the potential for CIs in preventing cccDNA formation *in vitro* (9, 10), although many mechanistic details behind such activity remain to be elucidated.

**FIG 1:**
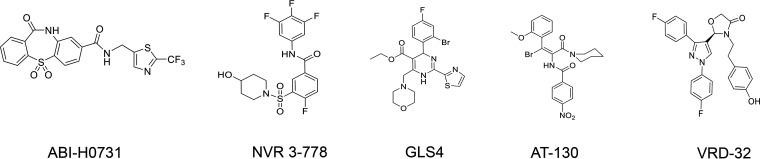
Chemical structures of CIs evaluated.

Multiple CIs have been previously disclosed (Fig. 1). ABI-H0731 is an HBV CI and a member of the novel dibenzo-thiazepin-2-one (DBT) chemical series. It is a direct-acting HBV antiviral whose mechanism of action consists primarily of interfering with HBV Cp oligomerization but inherently impacts many steps of the HBV life cycle. This study demonstrates that ABI-H0731 modulates the function of Cp by promoting the assembly of aberrant capsids, blocking the encapsidation of pgRNA, and inhibiting the subsequent replication of HBV DNA, which ultimately leads to the inhibition of the production of mature viral particles. Importantly, this study also establishes that ABI-H0731 can prevent cccDNA formation in *de novo* infection systems. The potency, selectivity, spectrum, and mechanism of action of ABI-H0731 and its basic drug-like properties were also evaluated in this preclinical study.

## RESULTS

### ABI-H0731 promotes the assembly of aberrant HBV capsids.

**FIG 2:**
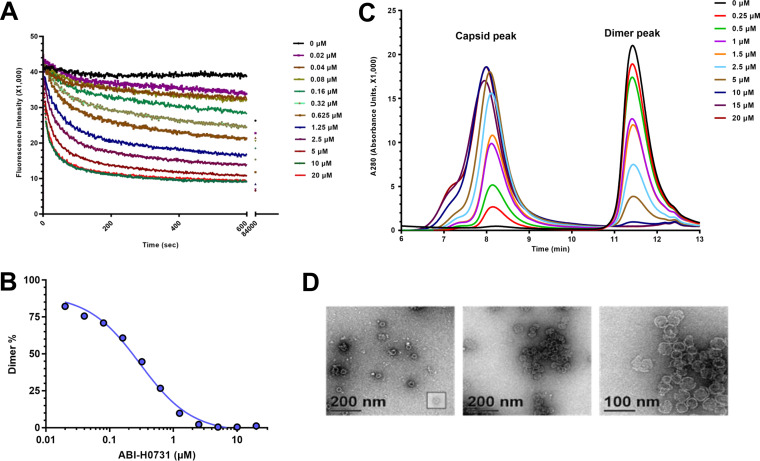
ABI-H0731 enhances the assembly of aberrant HBV capsids. (A) Effect of increasing concentrations of ABI-H0731 on the oligomerization of Cp150-BODIPY into capsids or other oligomeric structures through fluorescence quenching. The emission of fluorescence was monitored continuously for 1,400 min at 535 nm. (B) Nonlinear regression analysis of the free dimer levels as a function of the ABI-H0731 concentration. (C) Size exclusion chromatography (SEC) analysis of Cp assembly reactions performed in the presence of increasing concentrations of ABI-H0731. (D) Transmission electron microscopy (TEM) of Cp assembly reactions with DMSO (left) and 10 μM ABI-H0731 (middle and right).

The binding of CIs to HBV capsid subunits can lead to aberrant capsids by altering capsid assembly in different ways. Some CIs alter the morphology of HBV capsids, while others can enhance the formation of aberrant capsids or even promote their disassembly (11). The mechanism of action by which the CI ABI-H0731 interferes with the capsid assembly process was evaluated using a biochemical Cp assembly assay (12). In this assay, the N-terminal 150-residue assembly domain of Cp with a cysteine-to-alanine mutation (3CA Cp150) is linked to a BODIPY fluorophore that emits fluorescence when Cp150 is in a free dimer state. When Cp dimers oligomerize into capsids or other oligomeric structures, fluorophores become trapped inside the capsids and self-quench, causing a decline in fluorescence intensity. As shown in Fig. 2A, the incubation of purified Cp150 homodimers with ABI-H0731 resulted in a rapid decrease in the fluorescence intensity, indicative of a depletion of Cp150 free subunits and, inherently, the induction of Cp oligomerization. A 50% inhibitory concentration (IC_50_) of 267 nM was calculated by nonlinear regression analysis of the free dimer levels as a function of the ABI-H0731 concentration (Fig. 2B). Further analysis of the Cp assembly reactions by size exclusion chromatography (SEC) showed a decrease in the dimer peak (11.4 min) that was concomitantly accompanied in a dose-dependent manner by an increase in the capsid peak (8.2 min) (Fig. 2C). The shoulder on the capsid peak suggests the production of heterogeneous, large, noncapsid assembly products upon treatment with ABI-H0731. In line with the SEC data, transmission electron microscopy (TEM) showed that ABI-H0731 promotes the assembly of viral particles. At substoichiometric concentrations of ABI-H0731, assembly products appear to be outwardly normal 35-nm particles (Fig. 2D). However, large, aberrant spheroids become clearly visible with a high stoichiometric concentration of ABI-H0731 (10 μM). Overall, these results confirm that ABI-H0731 is a rapid and effective allosteric activator of HBV capsid assembly.

### ABI-H0731 inhibits HBV replication and cccDNA formation in cell culture.

The antiviral potency of ABI-H0731 was determined using two cell culture systems. The first is an HBV-inducible cell line (HepAD38) that bears a single “1.1 copy” of the HBV genome (genotype D, subtype ayw) under the control of a cytomegalovirus (CMV) tetracycline (Tet)-off promoter (13), and the second utilizes HBV infection of either a HepG2-derived cell line stably expressing the HBV entry receptor sodium taurocholate cotransporting polypeptide (NTCP) (HepG2-NTCP) or primary human hepatocytes (PHHs). The primary marker measured for inhibition of HBV replication in the cell culture systems is viral DNA. ABI-H0731 showed comparable inhibition of viral DNA replication in all three assay systems (HepAD38, HepG2-NTCP, and PHH cells), with 50% effective concentration (EC_50_) values of 173 nM, 307 nM, and 154 nM, respectively (Table 1), with the latter indicating good intracellular stability of ABI-H0731. In contrast, both GLS4 (14) and NVR 3-778 (7) suffered moderate to high viral load EC_50_ potency shifts in the PHH assay (see Table S1 in the supplemental material).

**TABLE 1:** ABI-H0731 potency in HBV cell culture models

Marker	Mean ABI-H0731 EC_50_ (nM) ± SD^*b*^		
	HepAD38	HepG2-NTCP	PHH
Viral DNA^*a*^	173 ± 40	307 ± 41	154 ± 18
HBeAg	NA	4,950 ± 520	2,210 ± 310
HBsAg	NA	7,300 ± 770	3,000 ± 270
pgRNA	NA	2,680 ± 580	1,840 ± 380

a Total intracellular viral DNA.

b NA, not applicable.

In HBV-infected HepG2-NTCP cells and PHHs, all viral moieties (viral DNA, pgRNA, and antigens) originate from viral infection, with pgRNA, HBeAg, and HBsAg acting as surrogate markers of cccDNA establishment and/or activity. This contrasts with the inducible HepAD38 assay system, in which viral pgRNA and antigens are expressed from the viral genetic material integrated into the host genome. ABI-H0731 demonstrated inhibition of pgRNA, HBeAg, and HBsAg production, with EC_50_s of 2.68 μM, 4.95 μM, and 7.30 μM, respectively (Table 1), in HBV-infected HepG2-NTCP cells. Similarly, pgRNA, HBeAg, and HBsAg levels in HBV-infected PHHs were inhibited by ABI-H0731, with EC_50_s of 1.84 μM, 2.21 μM, and 3.00 μM, respectively (Table 1). In the same experiment, two other CIs were also tested. GLS4 inhibits pgRNA, HBeAg, and HBsAg production, with EC_50_s of 0.28 μM, 0.55 μM, and 0.67 μM, respectively, but lost significant activity (1.93 μM, 1.48 μM, and 1.86 μM) in infected PHH cells (Table S1), likely due to its poor metabolic stability in hepatocytes. NVR 3-778 showed weaker activities in both infected cell systems. The results from both infection models clearly showed that ABI-H0731 interferes with cccDNA establishment and exhibits good stability in metabolically active PHHs (metabolic stability demonstrated by recovery of 67 to 84% of ABI-H0731 after 4 days in cultured PHH cells) (Fig. S1). In contrast, the NrtI entecavir (ETV) exhibits strong potency in inhibiting HBV replication in both infection models (EC_50_s of 0.8 nM and <0.1 nM, respectively), but it showed weak and incomplete inhibition of viral antigen production in HepG2-NTCP and PHH cells when tested at up to 100 nM (Table S1).

No cytotoxicity was observed in either of these HBV cell culture models at ABI-H0731 concentrations of up to 10 μM (data not shown). The effect of human serum proteins on ABI-H0731 potency was determined by evaluating whether there was a shift in potency when the assays were conducted in the presence of a high concentration of human platelet lysate (HPL) added to the culture medium. ABI-H0731 exhibited a modest loss of potency (≤2.6-fold) in the presence of 20% HPL (EC_50_, 522 ± 40 nM) compared to 2% HPL (EC_50_, 200 ± 5 nM) (Table S2).

**FIG 3:**
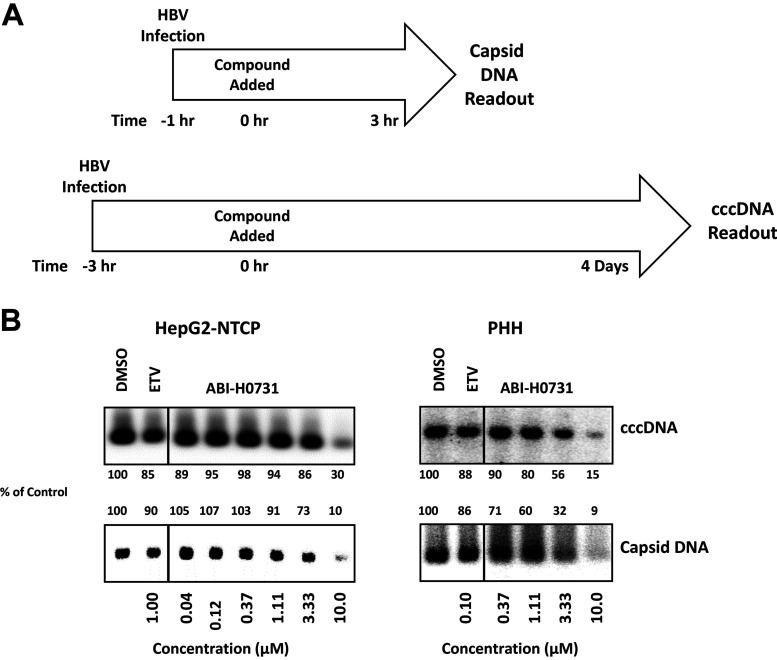
ABI-H0731 inhibits cccDNA establishment in *de novo*-infected cells. HBV infection assays in HepG2-NTCP cells and PHHs and Southern blot analysis of cccDNA and capsid DNA are described in Materials and Methods. (A) Treatment schedule. HepG2-NTCP cells and PHH cells were infected with HBV for 1 or 3 h for capsid DNA or cccDNA detection, respectively, and an MOI of 2,500 was used for capsid DNA analysis, whereas MOIs of 500 and 1,000 were used for cccDNA detection in HepG2-NTCP cell and PHH infection assays, respectively. After infection, the cells were treated with ABI-H0731 or ETV for 3 h or 4 days for capsid DNA or cccDNA detection, respectively. (B) Southern blot analysis of capsid DNA and cccDNA extracted from HBV-infected HepG2-NTCP cells and PHHs. Band density was quantified using ImageJ software.

To determine if ABI-H0731 specifically interferes with cccDNA formation, Southern blot analysis was performed on modified Hirt extracts from HBV-infected HepG2-NTCP and PHH cells that were treated with ABI-H0731 shortly after infection (Fig. 3A). These samples were subjected to T5 exonuclease digestion to degrade HBV relaxed circular DNA (rcDNA) and HBV double-stranded linear DNA (dslDNA), leaving the HBV cccDNA intact. HBV cccDNA was then linearized with the EcoRI endonuclease in order to generate a migration retardation pattern readily observable by Southern blotting using a radioactive probe specific for the HBV genome. Figure 3B (top left) shows that HBV cccDNA levels were significantly reduced when HepG2-NTCP cells were treated with concentrations approaching 10 μM ABI-H0731, 33× EC_50_ for viral replication (VR EC_50_), suggesting two distinct mechanisms, inhibition of nucleocapsid formation (and packaging of pgRNA) to prevent virion formation and premature melting of the incoming nucleocapsids to prevent trafficking to the nucleus and the subsequent generation of cccDNA. The latter mechanism requires significantly higher concentrations of CIs such as ABI-H0731 to demonstrate this activity. Other CIs also showed this dual antiviral mechanism, with much less potency in blocking cccDNA generation (10, 15). In contrast, levels of cccDNA in HepG2-NTCP cells treated with 1 μM ETV (>1,000× VR EC_50_) remained the same, indicating that CIs possess a unique inhibitory activity not found with NrtIs. This was further confirmed by the finding that ABI-H0731 inhibited cccDNA formation in PHHs at 3.3 μM and 10 μM (21× and 64× VR EC_50_s), whereas 100 nM ETV (>1,000× VR EC_50_) had no significant effect on cccDNA establishment in this HBV infection model (Fig. 3B, top right).

One possible mechanism of action by which ABI-H0731 could prevent cccDNA establishment in a *de novo* infection model involves the perturbance of the capsid’s integrity prior to the delivery of rcDNA to the nucleus. In such a scenario, a decrease in capsid DNA levels should be detected in ABI-H0731-treated cells following infection. Accordingly, the effects of increasing concentrations of ABI-H0731 on capsid DNA levels were evaluated and concomitantly compared to the levels of cccDNA formed. A significant decrease in capsid DNA levels was observed at 4 h postinfection in both infected HepG2-NTCP cells and PHHs (Fig. 3B, bottom), with cells treated with 10 μM ABI-H0731 for 3 h. This result appears to support a nucleocapsid-melting mechanism, as alternative mechanisms such as the stabilization of incoming capsids and the blocking of rcDNA transportation to the nucleus would instead be expected to increase capsid DNA levels when a CI is present.

Taken together, the direct assessments of cccDNA levels in both HepG2-NTCP and PHH infection models demonstrate that ABI-H0731 can prevent HBV cccDNA establishment in the context of a *de novo* infection model, confirming the results obtained using surrogate markers of cccDNA in these HBV infection models. While direct evidence of premature capsid melting by ABI-H0731 is difficult to demonstrate, the results obtained are supportive of the proposed mechanism that the blockage of cccDNA formation by ABI-H0731 is correlated with the ability of CIs to prematurely disrupt nucleocapsids and release viral DNA prior to trafficking to the nucleus of infected cells. It is important to note that while CIs can effectively prevent new cccDNA generation and establishment in infected cells, they appear to have no measurable effect on preexisting cccDNA pools.

### ABI-H0731 is an inhibitor of pgRNA encapsidation and rcDNA synthesis.

**FIG 4:**
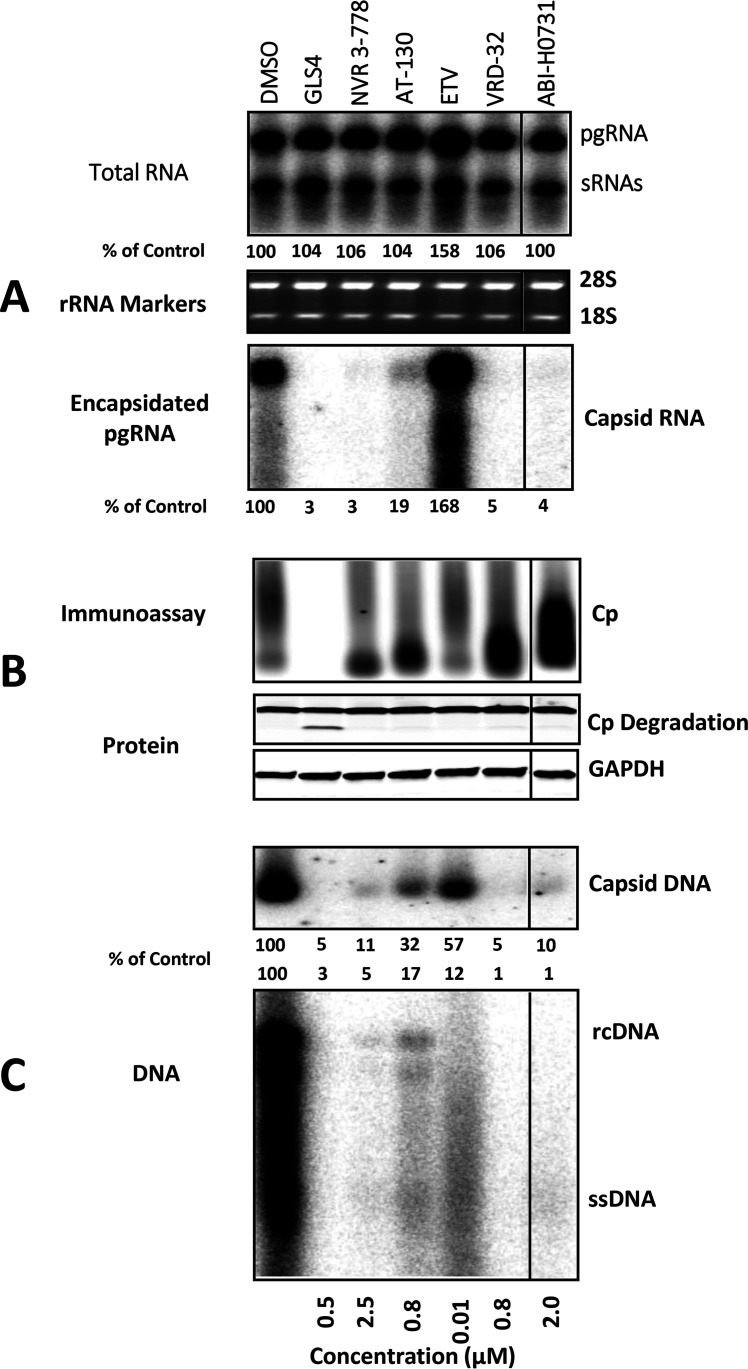
ABI-H0731 disrupts pgRNA encapsidation and HBV DNA replication. Shown are data from comparative analyses of the inhibitory effects of ABI-H0731, other CIs, and ETV at concentrations 10-fold over their VR EC_50_s on HBV infection markers of induced HepAD38 cells. (A) HBV pgRNA and surface RNAs (sRNAs) were detected from total RNA extracts via Northern blotting, with 18S and 28S rRNAs serving as loading controls. Levels of encapsidated RNA were detected from the micrococcal nuclease (MNase)-treated cell lysate by Northern blotting. (B, top) Disruption of capsid formation was examined in EIA gels. (Middle and bottom) Levels of HBV core protein (middle) and GAPDH (control) (bottom) were evaluated by Western blotting using total cell extracts. (C) Levels of total capsid-associated viral DNA (top) and the extracted HBV DNA replication intermediates (bottom) were assessed by Southern blotting. Band density was quantified using ImageJ software. rcDNA, relaxed circular DNA; ssDNA, single-stranded DNA.

Viral pgRNA must first be packaged into nascent capsids before its subsequent conversion to HBV DNA by the viral reverse transcriptase (RT) (3). Considering the ability of ABI-H0731 to interfere with the capsid formation process, pgRNA encapsidation was next evaluated in HepAD38 cells induced for 4 days while treated with ABI-H0731, other HBV CIs, as well as ETV. The CIs tested spanned diverse chemical series such as the heteroaryldihydropyrimidine (HAP) GLS4 (14), the sulfamoylbenzamide (SBA) NVR 3-78 (7, 11), the phenylpropenamide (PPA) AT-130 (16), the oxazolidinone VRD-32 (17), and ABI-H0731. The concentrations of all compounds were adjusted to 10-fold over their HepAD38 VR EC_50_s (data not shown). Following compound treatments, cells and culture medium were collected, and multiple markers of HBV infection were assessed. Northern blotting of total RNA with an HBV-specific probe showed that treatment with the various core inhibitors did not alter total intracellular HBV RNA levels (both pgRNA and HBs mRNAs) over the course of the experiment, as pgRNA expression was regulated by the Tet-off CMV promoter in HepAD38 cells (Fig. 4A, top). In cells treated with ETV, however, pgRNA levels appeared higher, a phenotype that was even more pronounced at the level of intracellular encapsidated pgRNA (Fig. 4A, bottom). Such stabilization of pgRNA can be attributed to the inhibition of the HBV RT/RNase H-mediated degradation of viral RNA, as reported previously (18). In sharp contrast to the NrtI inhibitory phenotype, all CIs, regardless of chemical series, potently blocked the encapsidation of pgRNA in HepAD38 cells. Levels of encapsidated pgRNA in cells treated with ABI-H0731, GLS4, VRD-32, and NVR 3-778 were virtually undetectable by Northern blotting (Fig. 4A, bottom). This inhibition of pgRNA encapsidation by ABI-H0731 and other CIs indeed translated into strong repression of HBV DNA replication, a process dependent on pgRNA encapsidation (3). The distinct mechanism of action of core inhibitors versus NrtIs was further demonstrated by Southern (HBV DNA) and Northern (pgRNA) blotting of secreted HBV viral particles in the supernatants (Fig. S2).

Cytoplasmic HBV capsid particles were resolved in a native agarose gel and analyzed by an HBV Cp enzyme immunoassay (EIA) (Fig. 4B, top). The results showed a heterogeneous variety of capsid migration patterns consistent with altered capsid sizes, as expected considering the mechanism of action of CIs, with GLS4-treated cells exhibiting an apparent loss of Cp. Immunoblotting (Fig. 4B, middle and bottom) using antibodies to Cp and glyceraldehyde-3-phosphate dehydrogenase (GAPDH) confirmed the selective degradation of Cp in GLS4-treated cells.

Southern blot experiments with an HBV-specific probe showed that the levels of encapsidated viral DNA and total intracellular viral DNA were both strongly reduced in cells treated with CIs compared to dimethyl sulfoxide (DMSO)-treated cells (Fig. 4C). Strikingly, rcDNA, the precursor of cccDNA, could not be detected in cells treated with ABI-H0731, GLS4, and VRD-32 under these experimental conditions. Treatment of HepAD38 cells with ETV prevented the formation of full-length rcDNA, but a significant amount of HBV DNA fragments could still be detected in capsids and total DNA extracts. This coincides with the mechanism of action of NrtIs, which cause chain termination during viral DNA synthesis, resulting in the generation and accumulation of shorter molecules of viral DNA.

The analysis of HepAD38 cell culture supernatants following treatment with CIs or ETV provided results that were virtually identical to the ones obtained with cell lysates. The quantification of extracellular HBV RNA and DNA levels by RT quantitative PCR (RT-qPCR) and qPCR, respectively, showed that the secretion of HBV RNA and DNA in the culture medium was strongly diminished in cells treated with ABI-H0731 (≤3% of the DMSO control) and other CIs (0.1 to 8.6% of the DMSO control) (Table 2). In contrast, ETV led to an increase in secreted levels of HBV RNA compared to DMSO-treated cells, while HBV DNA levels were still readily detectable albeit reduced by 80%. Taken together, these results demonstrated that ABI-H0731 efficiently blocks pgRNA encapsidation and the production of HBV RNA/DNA-containing particles, a phenotype that contrasts sharply with the stabilization of pgRNA and its enhanced secretion from cells treated with ETV.

**TABLE 2:** Extracellular DNA and pgRNA in HepAD38 cells

Compound	Concn (μM)	Extracellular DNA content (% of DMSO control)	Extracellular pgRNA content (% of DMSO control)
ETV	0.01	19.5	170
ABI-H0731	2.0	1.4	3.1
GLS4	0.5	0.1	0.1
NVR 3-778	2.5	1.0	2.0
AT-130	0.8	5.3	8.6
VRD-32	0.8	0.3	0.9

### The anti-HBV activity of ABI-H0731 is pangenotypic, highly selective, and noncytotoxic.

An effective anti-HBV therapeutic intervention should display activity against several highly seroprevalent genotypes, such as genotypes A, B, C, and D. Hence, the activity of ABI-H0731 was profiled in cells transiently expressing representative genomic sequences of HBV strains from all four above-listed genotypes. All four strains utilized in this study exhibited good replication fitness in cell culture systems (data not shown). As shown in Table 3, ABI-H0731 showed broad-spectrum activity against all four of the genotypes tested, with VR EC_50_s ranging from 86 to 142 nM. The 142 nM VR EC_50_ observed in cells transiently expressing an HBV genotype D (Gt-D) construct was highly similar to the 173 nM VR EC_50_ observed in the HBV-inducible HepAD38 cell (Gt-D) culture system. Used as an assay control, the pangenotypic NrtI ETV exhibited VR EC_50_s ranging from 1.2 to 5.0 nM across all four genotypes (Table 3). All 4 HBV genotypes exhibited good replicative fitness as determined by Southern blot analysis (Fig. S3).

**TABLE 3:** ABI-H0731 activity against HBV genotypes A to D

HBV genotype	Mean antiviral EC_50_ (nM) ± SD	
	ABI-H0731	ETV
A	113 ± 22	1.9 ± 0.5
B	85.5 ± 10.9	2.3 ± 0.5
C	88.2 ± 27.0	1.2 ± 0.1
D	142 ± 48	5.0 ± 1.9

HBV Cp is highly conserved and has no homology to other viral core proteins. ABI-H0731 specifically binds to a unique dimer-dimer interface formed by HBV Cps. In order to determine whether the potency observed with ABI-H0731 is selective for HBV in cells, cell protection assays were performed with 3 unrelated viruses: herpes simplex virus 1, a double-stranded DNA virus; human rhinovirus 16, a positive-stranded RNA virus; and influenza A virus, a negative-stranded RNA virus. ABI-H0731 showed no significant inhibitory activity against any of these viruses at concentrations of up to 10 μM (data not shown).

Cytotoxicity assays are useful for assessing whether the antiviral activity observed in cell culture studies is in fact due to nonselective inhibition of cellular functions required for viral replication. ABI-H0731 was evaluated for cytotoxicity by monitoring the viability of seven human cell types/lines (Huh7, HepG2, HeLa H1A, HEK293, NCI-H226, MOLT-4, and freshly isolated peripheral blood mononuclear cell [PBMC] cultures representing various human tissues) incubated for 4 days with various concentrations of ABI-H0731. At the highest concentrations tested (10 μM), ABI-H0731 showed no substantial reduction in cell viability in any of the cell types/lines tested, resulting in 50% cytotoxic concentration (CC_50_) values of >10 μM (data not shown), a value >50-fold higher than the VR EC_50_ of ABI-H0731. Collectively, these results confirm that the inhibitory activity of ABI-H0731 is pangenotypic while being highly specific and selective for HBV.

### ABI-H0731 resistance profile.

Viral resistance often emerges with the prolonged use of NrtIs that possess a low resistance barrier, such as lamivudine (3TC), resulting in high viral relapse rates in patients. It was therefore important to determine whether there is any loss in ABI-H0731 potency against strains harboring NrtI resistance (NrtI^R^) mutations. Accordingly, an inducible NrtI^R^ stable cell line (HepG2-NrtI^R^) harboring the signature 3TC resistance substitutions (rtL180M/M204V) was established. The expression of this NrtI^R^ virus and its loss of susceptibility to ETV were characterized. *In vitro* analysis of qPCR and Southern blot data demonstrated that the rtL180M/M204V mutant strain displayed a 10- to 12-fold shift in susceptibility to ETV compared with the wild-type virus (Table 4 and Fig. S4), which was similar to previous results with other 3TC-resistant strains (rtL180Q/M204V) (19). The NrtI^R^ cells were treated for 4 days with ABI-H0731 or ETV immediately following induction. The VR EC_50_ of ABI-H0731 in HepG2-NrtI^R^ cells was 262 nM, which is similar to the VR EC_50_ of 221 nM concomitantly determined in HepAD38 cells (Table 4). In contrast, the VR EC_50_ of ETV increased by over 12-fold, from 0.6 nM in HepAD38 cells to 7.1 nM in HepG2-NrtI^R^ cells, a loss of potency consistent with what has been reported previously for this set of NrtI^R^ mutations (20).

**TABLE 4:** ABI-H0731 is active against NrtI^R^ HBV

Mean ABI-H0731 EC_50_ (nM) ± SD		Fold shift for ABI-H0731	Mean ETV EC_50_ (nM) ± SD		Fold shift for ETV
HepG2-NrtI^R^	HepAD38		HepG2-NrtI^R^	HepAD38	
262 ± 97	221 ± 19	1.2	7.1 ± 1.2	0.58 ± 0.15	12.3

Amino acid substitutions in Cp found in clinical isolates have already been reported to impact the antiviral activity of specific chemical series of CIs, with some mutations causing resistance to specific chemical classes (7, 11, 21, 22). To probe potential resistance substitutions as well as gain valuable insight into the critical amino acids at the ABI-H0731 binding site, a panel of HBV expression vectors carrying representative single mutations in the binding pocket of Cp was generated. These constructs were transiently transfected into Huh7 cells, and resistance to ABI-H0731 was evaluated and compared to the potency of other CIs from three other chemical series: HAPs, SBAs, and oxazolidinones. The results showed that the antiviral activity of ABI-H0731 was reduced by 20-, 21-, and 14-fold against variants with D29G, T33N, and Y118F substitutions, respectively (Table 5). The T33N substitution appears to decrease susceptibility to all CIs tested, regardless of chemical class, with the antiviral activities of NVR 3-778, GLS4, and VRD-32 decreasing by >56-fold, 350-fold, and 80-fold, respectively. In contrast, only specific CI chemical series exhibited significant resistance to the D29G and Y118F variants. ABI-H0731 exhibited the greatest loss of potency against the T109I and T109M variants, with a loss of antiviral activity of >68-fold. ABI-H0731 retained full potency against the P25A substitution, a variant that results in a 25-fold loss of GLS4 potency. ETV was used as a negative control and did not exhibit a significant potency shift against any of the variants tested.

**TABLE 5:** Activity of ABI-H0731 against HBV Cp variants

HBV Cp substitution	Clinical isolate frequency (%)^*a*^	Fitness (%)	ABI-H0731		NVR 3-778		GLS4		VRD-32		ETV	
			Mean EC_50_ (nM) ± SD	Fold shift	Mean EC_50_ (nM) ± SD	Fold shift	Mean EC_50_ (nM) ± SD	Fold shift	Mean EC_50_ (nM) ± SD	Fold shift	Mean EC_50_ (nM) ± SD	Fold shift
Wild type		100	146 ± 21	1	178 ± 42	1	17.9 ± 0.4	1	16.1 ± 7.8	1	1.9 ± 0.9	1
P25A	<0.1	122	169 ± 41	1.2	719 ± 58	4	451 ± 3.3	25	15.9 ± 0.04	1	2.9 ± 0.3	1.5
D29G	0.1	19	2,961 ± 917	20	210 ± 52	1.2	16.1 ± 0.4	0.9	13.8 ± 1	0.9	0.6 ± 0.1	0.3
T33N	<0.1	61	3,061 ± 187	21	>10,000	>56	6,345 ± 2,448	350	1,289 ± 242	80	3.5 ± 1.6	1.8
Y38C	0.1	92	224 ± 50	1.5	150 ± 48	0.8	17 ± 9.5	0.9	20 ± 1.4	1.2	1.1 ± 0.5	0.6
Y38F	3.2	55	481 ± 34	3.3	208 ± 49	1.2	65 ± 17	3.7	20 ± 10	1.3	4.6 ± 0.6	2.4
I105L	0.7	73	41 ± 6	0.3	127 ± 73	0.7	12 ± 6.5	0.6	40 ± 19	2.5	0.4 ± 0.2	0.2
I105T	0.6	52	324 ± 20	2.2	726 ± 98	4.1	17 ± 2.7	0.9	103 ± 87	6.4	0.5 ± 0.3	0.4
T109I	0.2	52	>10,000	>68	77.4 ± 0.4	0.4	237 ± 16.6	13	15.8 ± 1.9	1	1.9 ± 0.6	1.0
T109M	0.8	29	>10,000	>68	161 ± 97	0.9	15 ± 5.6	0.9	25 ± 23	1.6	1.5 ± 0.3	0.8
Y118F	0.4	9	2,070 ± 264	14	887 ± 478	5	84.7 ± 2.3	4.7	805 ± 423	50	1.5 ± 0.9	0.8

a See https://hbvdb.lyon.inserm.fr/HBVdb/.

The replicative fitnesses of all constructs presented in Table 4 were between 9% and 122% of that of the wild-type control. HBV constructs bearing other variants were also created; however, their replication fitness was too low to enable the generation of reproducible data (data not shown). A review of the available HBV sequence databases (https://hbvdb.lyon.inserm.fr/HBVdb/) indicates that except for the Y38F variant (3.2%), none of the substitutions studied preexist as a natural polymorph in >1% of patient isolates (Table 5). Currently, multiple classes of CIs are still in early-stage clinical development (phases 1 and 2) (23, 24), and clinical baseline polymorphisms and on-treatment enrichment of substitutions reducing the *in vitro* activity of CIs are rare, with a few impacting the virological response (23, 24). In the phase 1b study ABI-H0731-101(B), there was one HBeAg-negative patient who exhibited only a 1.0-log decline by day 28 on monotherapy, who was subsequently found to harbor a known CI-resistant variant (T109M) at baseline. Population sequencing results showed that this substitution was present in 70% of the circulating virus population at the time of study entry, with a subsequent increase to 100% by day 8 of monotherapy treatment. No other resistance mutations developed during the study. As longer treatment times in phase 2 studies utilize combination therapy (CI plus NrtI), resistance has been very rare and only found to be associated with treatment noncompliance.

### HBV replication is strongly inhibited by the combination of ABI-H0731 and ETV.

Drug combination testing has been used effectively to evaluate interactions between inhibitors from distinct mechanistic classes. HBV replication was thus assayed in the presence of both ABI-H0731 and ETV to determine whether such a drug combination was additive, synergistic, or antagonistic. One of the two methodologies used relied on MacSynergy II analysis (25), which calculates antagonism and synergy volumes for drug combinations. Drugs that have antagonism volumes of less than −25 μM^2^% or synergy volumes of more than 25 μM^2^% are considered antagonistic or synergistic, respectively; otherwise, they are considered additive. Results from this analysis are summarized in Table 6 and demonstrate that the combination of ABI-H0731 and ETV in the HepAD38 cell culture system was additive, with negligible synergy and antagonism volumes of 0.56 μM^2^% and −1.57 μM^2^% (95% confidence interval), respectively. Drug interactions were also be evaluated by a second methodology that features the combination index (CI) calculation as described previously by Chou and Talalay (26). The CI can indicate synergism (CI < 0.8), an additive effect (CI = 0.8 to 1.2), or antagonism (CI > 1.2). CI values calculated for VR EC_50_, EC_75_, and EC_90_ values of paired ABI-H0731 and ETV were 1.09 (additive), 0.75 (slightly synergistic), and 0.53 (synergistic), respectively (Table 7). Thus, both analytical approaches indicated that ABI-H0731 and an NrtI have an additive to moderately synergistic effect. More importantly, no antagonism was observed at any concentrations using either methodology.

**TABLE 6:** Results of combination studies using MacSynergy II

Interaction	Log vol	Vol (μM^2^%)	Interpretation
Synergy	0.18	0.56	Additivity (insignificant synergy)
Antagonism	−0.49	−1.57	Additivity (insignificant antagonism)

**TABLE 7:** ABI-H0731 combination index (CompuSyn) results

Combination (ratio)	Mean CI ± SD				Interpretation
	EC_50_	EC_75_	EC_90_	Overall	
ETV (1:160)	1.09 ± 0.19	0.75 ± 0.09	0.53 ± 0.06	0.79 ± 0.11	Moderate synergy

**FIG 5:**
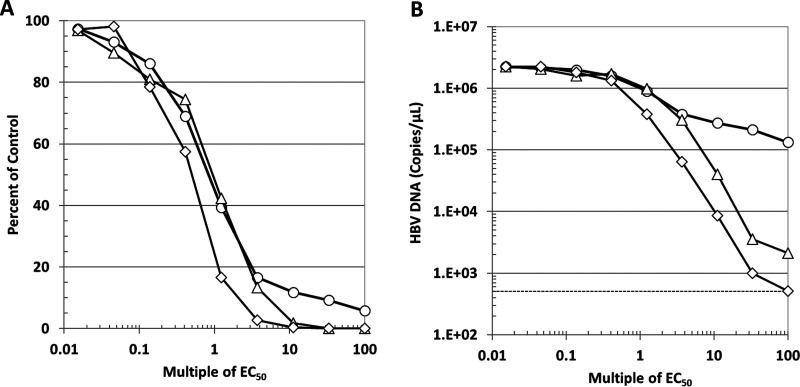
The combination of ABI-H0731 and ETV enhances the suppression of HBV replication. Induced HepAD38 cells were treated for 4 days with the indicated ranges of concentrations of ABI-H0731 and ETV, either alone or in combination. Viral replication was evaluated by quantifying the levels of total HBV DNA in the cells by qPCR. Results presented are percentages of DMSO-treated cells (A) or absolute levels of HBV DNA in genome copies per microliter (B). In both cases, the data are plotted as a function of each drug’s concentration relative to its VR EC_50_. Circle, ETV; triangle, ABI-H0731; diamond, ABI-H073+ETV.

To further examine the inhibitory effect of combining ETV and ABI-H0731 on HBV replication, induced HepAD38 cells were subjected to 4-day treatments with both drugs, alone and in combination, at matching concentrations that spanned 0.015× to 100× VR EC_50_. Traditional dose-response curves, as depicted in Fig. 5A, showed that the inhibition of HBV replication in HepAD38 cells starts to plateau when the concentration of ETV reaches 3.7× VR EC_50_ (3.7 nM). At 100× VR EC_50_ of ETV (100 nM), the inhibition of HBV replication had not yet reached 95%, with HBV DNA levels being about 5% of those of the DMSO control (no drug). In sharp contrast, inhibition of HBV replication by ABI-H0731 reached 98% at 10× VR EC_50_ (1.7 μM). Most encouraging was that the combination of both drugs allowed for a faster and deeper decline at lower concentrations of both inhibitors, with <4× VR EC_50_s of both inhibitors reducing HBV DNA levels by >97%. The enhanced inhibition of HBV replication through the combination of both drugs became striking when the absolute numbers of HBV genome copies were plotted instead of values relative to the DMSO control. As shown in Fig. 5B, 100× VR EC_50_ of ETV only reduced HBV DNA levels from 2.2 × 10^6^ to 1.3 × 10^5^ copies/μl (∼1 log), whereas ABI-H0731 had already inhibited HBV DNA replication by ∼3 logs (2.1 × 10^3^ copies/μl) at 60× VR EC_50_ (10 μM, the maximum concentration of ABI-H0731 tested). Remarkably, the treatment of HepAD38 with the combination of both ETV and ABI-H0731 further reduced viral replication compared to cells treated with ABI-H0731 alone at all concentrations starting at 1× VR EC_50_. At the highest doses (100 nM ETV and 10 μM ABI-H0731), the levels of HBV DNA had in fact dropped to the background levels of the assay, as determined by the quantification of HBV DNA from uninduced HepAD38 cells (Fig. 5B, dotted line). Taken together, these results underscore that the combination of ETV and ABI-H0731 does not exhibit antagonistic effects and that, on the contrary, such a combination has the potential to reduce HBV replication to levels that are lower than those when either drug is used alone.

### ADME properties.

The inhibition of CYP1A2, -2B6, -2C8, -2C9, -2C19, -2D6, and -3A isozymes was explored over an ABI-H0731 concentration range of 0.1 to 100 μM. CYP IC_50_ values ranged from 27.2 μM for 2C8 to >100 μM for 1A2 (Table 8). The potential for ABI-H0731 to activate pregnane X receptor (PXR) and induce CYP3A4 was investigated in DPX2 cells harboring the human PXR gene (NR1I2) and a luciferase reporter gene linked to two CYP3A4 gene promoters. When cells were incubated with 10 and 40 μM ABI-H0731 for 24 h, PXR activation activities were 29% and 68% of the control activity, respectively.

**TABLE 8:** ABI-H0731 inhibition of major CYP isoforms in human liver microsomes

Test article	Test concn (μM)	CYP IC_50_ (μM)							
		1A2	2B6	2C8	2C9	2C19	2D6	3A (MDZ^*a*^)	3A (test)
ABI-H0731	0.1–100	>100	27.7	27.2	30.7	42.8	29.6	82.3	46.2

a MDZ, midazolam (CYP3A substrate).

The *in vitro* stability of ABI-H0731 was investigated using liver microsomes and hepatocytes from humans, mice (microsomes only), rats, dogs, and cynomolgus monkeys to model the hepatic clearance rate of ABI-H0731. Predicted clearance rates (CL_pred_) and *in vitro* half-life (*T*_1/2_) values are presented in Table 9. ABI-H0731 is stable in human, mouse, rat, and monkey microsomes, with CL_pred_ values ranging between 12% and 21% of liver blood flow in these species. Dogs exhibited less stability than the other species tested, resulting in intrinsic clearance values 3 to 5 times higher than those for the other species tested. Hepatocyte data were consistent with microsome data. ABI-H0731 is stable in human, rat, and monkey hepatocytes (CL_pred_ ranging from 2.3 to 21 ml/min/kg), corresponding to a range of 11% to 32% of liver blood flow. Similar to the trend in liver microsomes, ABI-H0731 was extensively metabolized in dog hepatocytes (CL_pred_ = 25 ml/min/kg), compared to the other species tested.

**TABLE 9:** ABI-H0731 cross-species liver microsome and hepatocyte stability^*a*^

Species	Microsomes			Hepatocytes		
	CL_pred_ (ml/min/kg)	*T*_1/2_ (min)	% LBF	CL_pred_ (ml/min/kg)	*T*_1/2_ (min)	% LBF
Human	2.8	376	13	2.3	695	11
Mouse	19	140	21	ND	ND	ND
Rat	7.9	277	12	21	105	32
Dog	20	37.3	65	25	40	81
Monkey	4.7	359	15	4.7	475	15

a ND, not determined; %LBF, percentage of liver blood flow.

Despite exhibiting a <3-fold loss in efficacy in the presence of human serum, ABI-H0731 is highly bound to plasma proteins in human, monkey, rat, and mouse. In human, ABI-H0731 was measured to be 96.9%, 96.6%, and 97.6% bound at 3, 10, and 30 μM, respectively. The extents of plasma protein binding in mouse, rat, and monkey plasma were similar to that seen in human plasma, with ranges of 96.6% to 97.0%, 94.8% to 98.0%, and 97.6% to 96.4%, respectively, over the concentration range of 3 to 30 μM. There were no apparent changes in the extent of binding over the concentration ranges explored in any species.

### *In vivo* pharmacokinetics of ABI-H0731.

Overall, *in vitro* clearance data from microsomes and hepatocytes were aligned with *in vivo* clearance results, where low clearance was observed in mice, rats, and monkeys and higher clearance was observed in dogs (Table 10). ABI-H0731 was formulated as a solution and administered via intravenous (i.v.) injection into CD-1 mice, Sprague-Dawley rats, beagle dogs, and cynomolgus monkeys in order to obtain intrinsic pharmacokinetic parameters. The same solution formulation was administered *per os* (p.o.) in the same species to determine oral bioavailability. After i.v. administration, the drug rapidly distributed and decayed in a biphasic manner in all species tested. Plasma clearance values of ABI-H0731 were 8.05, 10.1, 14.7, and 4.86 ml/min/kg, representing 9, 16, 47, and 11% of liver blood flow, in mouse, rat, dog, and monkey, respectively. The volume of distribution was moderate, ranging between 1.56 and 4.06 liters/kg, indicating ABI-H0731 distribution outside the plasma compartment. The terminal half-life values after i.v. administration were 3.6, 4.9, 2.5, and 12.6 h in mouse, rat, dog, and monkey, respectively. After p.o. administration, ABI-H0731 was well absorbed, with oral bioavailability (%*F*) ranging from 49% in dogs to 95% in rats (Table 10). Maximum plasma exposures were observed at between 0.7 and 3.3 h (time to maximum concentration of drug in serum [*T*_max_]). Given that the target of ABI-H0731 administration is the liver (27, 28), 24-h trough liver ABI-H0731 concentrations were determined after repeat-dose administration in rat and monkey. Consistent with the moderate volume of distribution, liver-to-plasma ratios are 13 and 26 in rat and monkey, respectively. Finally, ABI-H0731 exhibited no specific target organ or dose-limiting toxicities in an extensive panel of toxicology studies (data not shown) and has also been well tolerated in human clinical studies (29, 30).

**TABLE 10:** ABI-H0731 pharmacokinetics in mouse, rat, dog, and monkey after single-dose i.v. or oral solution administration^*a*^

Species^*b*^	Route^*c*^	Dose (mg/kg)	*C*_max_ (ng/ml)	*T*_max_ (h)	AUC_inf_ (ng · h/ml)	CL (ml/min/kg)	*V*_ss_ (liters/kg)	*T*_1/2_ (h)	%*F*
Mouse	i.v.	1			2,070	8.05	2.16	3.55	84
	p.o.	5	1,420	1.00	8,690			3.03	

Rat	i.v.	1			1,670	10.1	3.47	4.92	95
	p.o.	2	324	1.00	3,160			6.13	

Dog	i.v.	2			2,890	14.7	1.56	2.46	49
	p.o.	3	861	0.667	2,150			3.34	

Monkey	i.v.	0.5			1,750	4.86	4.06	12.6	55
	p.o.	2	353	3.33	4,340			11.6	

a *C*_max_, maximum concentration of drug in serum; AUC_inf_, area under the concentration-time curve from time zero to infinity; *V*_ss_, volume of distribution at steady state.

b For mice, a composite study design was utilized (*n* = 3/time point/dose group). In other species, individual animals were sampled serially (*n* = 3/dose group).

c For intravenous (i.v.) and oral (p.o.) administration, ABI-H0731 was formulated in a solution containing 5% (vol/vol) NMP–5% (vol/vol) Solutol HS-15–90% (vol/vol) normal saline.

## DISCUSSION

HBV Cp is critical to many aspects of the HBV life cycle. This is underscored by several studies demonstrating that the inhibition of Cp perturbs biological processes such as viral capsid assembly, viral RNA encapsidation, viral DNA replication, HBV cccDNA establishment and transcription, as well as host immune responses to HBV infection (6, 7, 9–11, 14, 16). As a result, CIs have become a promising class of new anti-HBV therapeutics, with several candidates already in human clinical trials for the treatment of CHB patients (23, 31). Here, we report the preclinical profiling of ABI-H0731, a novel and effective HBV CI currently in phase 2a human clinical trials (29, 30). ABI-H0731 was evaluated for its ability to impact viral capsid assembly, prevent viral RNA encapsidation and HBV DNA replication, and interfere with cccDNA establishment. The HBV genotype coverage of ABI-H0731 and its selectivity, cytotoxicity, resistance, mechanism of action, and drug-like properties were also assessed, along with its activity interaction with ETV in drug combination studies.

Biochemical data demonstrate that ABI-H0731 profoundly accelerates the oligomerization of free Cp dimers into capsids and promotes the formation of aberrant oligomeric structures at high concentrations. ABI-H0731 was able to potently inhibit HBV DNA replication in three cell culture models, the HepAD38 inducible system along with infection of HepG2-NTCP cells and PHHs. In contrast to ETV treatment, ABI-H0731 also significantly decreased the levels of the key surrogate markers of cccDNA (pgRNA, HBeAg, and HBsAg) in infected HepG2-NTCP cells and PHHs, a setting in which these HBV markers are solely derived from newly established cccDNA molecules. In addition, the data presented also showed that this phenotype correlated well with the rapid decrease in the levels of cytoplasmic capsid DNA in these newly infected cells. One could hypothesize that ABI-H0731 promotes the premature release of incoming encapsidated rcDNA, perturbing its subsequent delivery to the nucleus and, ultimately, the formation of cccDNA. A mechanism of action involving such a premature cytoplasmic exposure of capsid rcDNA has been proposed for GLS4 from the HAP series (32). Nevertheless, more studies are required to confirm the mechanistic details behind the inhibition of cccDNA generation exhibited by ABI-H0731. CIs are the only class of CHB therapeutics that have been shown to inhibit the surrogate markers of cccDNA formation in HBV-infected cells (9, 10), as demonstrated by ABI-H0731, and not ETV, in *de novo*-infected HepG2-NTCP cells and PHHs. Thus, one could envision that in a clinical setting, CIs would directly interfere with HBV reinfection and persistence in CHB patients via the blockage of cccDNA establishment. Such a therapeutic effect would greatly improve cure rates compared to the current standard of care, NrtI therapy.

The divergence of anti-HBV activity between ETV and ABI-H0731 was further illustrated when the levels of encapsidated viral RNA were investigated. Induced HepAD38 cells treated with ABI-H0731 had dramatic reductions in the levels of encapsidated pgRNA, intracellular and extracellular, whereas ETV had the opposite effect. This can be attributed to their distinct mechanisms of action. ABI-H0731 perturbs capsid formation and pgRNA recruitment, whereas ETV stalls the reverse transcription process, thus preventing the degradation of pgRNA. The observed increase in the release of HBV RNA in the culture medium of ETV-treated cells was in line with data reported in previous studies, including a study by Wang et al., who showed that pgRNA leakage was associated with the persistence of viral infection and rebound (18). Accordingly, the ability of ABI-H0731 to dramatically reduce the release of pgRNA in the extracellular milieu would eliminate such viral persistence concerns inherent in the mechanism of action of NrtIs.

Since CIs from different chemical classes do not share the same contact points in the Cp dimer-dimer pocket, they would therefore be expected to exhibit only a partial overlap when profiled against a panel of binding site variants. Accordingly, key amino acid substitutions known to reside at the interface of the CI binding pocket confer resistance levels related to how individual CIs are oriented in the binding pocket. Significant cross-resistance to all CIs is observed when T33N and Y118F substitutions are present, while more selective resistance is noted with substitutions such as G29G and Y109I/M (ABI-H0731) and P25A (GLS4). Fortunately, the level of preexistence of these putative resistant variants is very low (<1%) in the overall HBV patient population based on analyses of public database sequences of clinical isolates (Table 5). Moreover, it is anticipated that the clinical use of CIs will always be in combination with NrtI therapy in order to maintain an exceedingly high resistance barrier and prevent the enrichment of any CI-resistant variants with prolonged therapy. A preexisting T109M variant was discovered at baseline in a patient treated with ABI-H0731 in a phase 1b 28-day monotherapy clinical study (23). Although *in vitro* analysis showed that ABI-H0731 susceptibility was diminished >68-fold, this patient still exhibited a 1-log decline in viral load by day 28. Nevertheless, it will be important to monitor the frequency and potential emergence of these Cp variants in ongoing clinical trials featuring CIs in combination with NrtI therapy.

An effective HBV antiviral should display activity against a majority of highly seroprevalent genotypes, especially considering the regional differences in HBV epidemiology worldwide. ABI-H0731 showed broad-spectrum activity against representative sequences of genotypes A, B, C, and D, the most prevalent genotypes globally (33). This broad-spectrum activity should not come as a surprise considering the high level of conservation of residues at the binding site of CIs (5) as well as those specifically interacting with ABI-H0731 as defined in this study.

Combinations of antiviral agents that inhibit viral replication by different mechanisms can significantly improve efficacy and durability for the treatment of chronic virus infections, as already established for human immunodeficiency virus (HIV) or hepatitis C virus (HCV) infections (34, 35). Combination studies conducted with ABI-H0731 and ETV demonstrated their additivity and lack of antagonism as well as their potential synergistic activity at higher doses. Remarkably, the combination of ABI-H0731 with the NrtI ETV resulted in a more sustained suppression of HBV DNA replication in which viral DNA levels in induced HepAD38 cells became indistinguishable from the background levels in the assay. Such a level of inhibition could not be achieved with either drug alone, even with high doses. One could envision that the ability of ABI-H0731 to enhance the inhibition of HBV replication in the presence of NrtIs could translate into better suppression of HBV in CHB patients, hopefully enhancing inhibitory efficacy and significantly improving cure rates. The outcome of ongoing phase 2a clinical trials studying the effect of ABI-H0731 in combination with NrtIs in HBV chronically infected patients should shed light on these questions (29, 30).

Taken together, the potency, unique mechanism of action, drug-like properties, and favorable preclinical safety profile support the continued clinical development of ABI-H0731.

## MATERIALS AND METHODS

### HBV Cp *in vitro* assembly assays.

HBV Cp *in vitro* assembly assays were conducted as previously described (12, 36, 37). Details are as follows.

### (i) Sample preparation.

Core protein truncation constructs containing the assembly domain from HBV subtype adyw, either wild-type Cp149 or its cysteine-to-alanine mutant 3CA Cp150, were expressed in Escherichia coli from pET11-based plasmids and purified by size exclusion coupled with capsid disassembly as described previously (12, 36, 37).

### (ii) Protein BODIPY labeling.

3CA Cp150 BODIPY (4'4-difluoro-4-bora-3a,4a-diaza-S-indacene) dye labeling was carried out as previously described (37). Briefly, prior to labeling, dithiothreitol (DTT) was removed from the protein stock by using a PD-10 desalting column equilibrated with ice-cold 50 mM HEPES (pH 7.5). Fractions were pooled by measuring the protein concentration at 280 nm, a 20-fold molar excess of BODIPY-maleimide was then mixed with protein, and the mixture was incubated at 4°C overnight. On the second day, DMSO and unreacted free BODIPY were removed by PD-10 desalting.

### (iii) Fluorescence quenching assay.

The HBV capsid assembly assay was monitored by fluorescence quenching in a plate reader (BioTek H1) in a black 96-well nonbinding plate (Greiner). The assembly reaction was performed at room temperature with 1.2 μM Cp150-BODIPY, 400 mM NaCl, and ABI-H0731 from 0 to 20 μM. ABI-H0731 at various concentrations was preincubated with Cp150-BODIPY for 1 min prior to inducing assembly with salt. Fluorescence measurements were initiated immediately after the addition of salt by a dispenser. The plate was sealed after a continuous 10-min kinetic reading and then left in the dark overnight at room temperature. After 24 h of incubation, endpoint fluorescence was recorded.

### (iv) Size exclusion chromatography.

Frozen aliquots of the HBV Cp assembly domain truncation mutant Cp149 were dialyzed into 50 mM HEPES (pH 7.5). Protein was incubated with the compound for 1 min prior to assembly induction by the addition of 5 M NaCl to final concentrations of 5 μM Cp, 1% DMSO, and 150 mM NaCl. Reaction mixtures were incubated at 37°C for 24 h, and the reactants and products were then separated by high-performance liquid chromatography (HPLC) (Shimadzu) with a 4.6- by 150-mm Bio SEC5 1,000-Å column in tandem with a 4.6- by 150-mm Bio SEC5 300-Å column. Reactions were run at 37°C in a solution containing 50 mM HEPES and 150 mM NaCl at 0.5 ml/min. Reactants and products were quantified using the LabSolutions software package (Shimadzu).

### (v) Electron microscopy.

Samples from SEC experiments were adsorbed to glow-discharged carbon over parlodion carbon-coated copper grids (EM Sciences). Samples were negatively stained with 0.75% uranyl formate and visualized with a JEOL 1010 transmission electron microscope equipped with a 1K×1K Gatan charge-coupled-device (CCD) camera.

### Cell culture.

Growth medium for Huh7 and HepG2 cells contained Dulbecco’s modified Eagle’s medium (DMEM) supplemented with 10% heat-inactivated fetal bovine serum (FBS) and 1× Pen/Strep (100 IU/ml penicillin and 100 μg/ml streptomycin). HepAD38 growth medium is composed of DMEM and Ham’s F-12 nutrient medium in a 1:1 mixture, supplemented with 10% FBS, 1× Pen/Strep, 250 μg/ml of G418, and 1 μg/ml of tetracycline (Tet). HepAD38 induction medium consists of DMEM–F-12 medium supplemented with 2% Tet-system-approved FBS (Tet free) and 1× Pen/Strep. HepG2 cells stably expressing sodium taurocholate cotransporting polypeptide (NTCP) were grown in HepG2-NTCP growth medium composed of DMEM growth medium supplemented with 125 μg/ml G418. Primary human hepatocytes (PHHs) (TRL) were cultured in PHH maintenance medium, which consists of Williams E medium supplemented with cell maintenance cocktail B (Gibco). HepG2-NTCP cells and PHHs were both cultured in vessels coated with 50 μg/ml rat tail collagen.

### Compounds.

ABI-H0731 was synthesized and purified under contract at Sai Life (India). Other chemical compounds were obtained from commercial sources.

### HBV replication assay in HepAD38 cells.

HepAD38 cells were trypsinized and then washed three times with phosphate-buffered saline (PBS) to remove traces of Tet. Cells were seeded into 96-well plates in assay medium (DMEM–F-12 medium with 2% Tet-free FBS) and treated with compounds. Supernatants and cells were harvested at 5 days postinduction. Viral loads in supernatants and cells were quantified by TaqMan qPCR using a primer pair (5′-CTGTGCCTTGGGTGGCTTT-3′ and 5′-AAGGAAAGAAGTCAGAAGGCAAAA-3′) and a probe (5′-5HEX-CTCCACAGT-ZEN-AGCTCCAAATTCTTTATAAGGGTC-3IABkPQ-3′) specific for the core gene sequence.

### HBV purification, infection, and compound treatment.

HBV was purified from the culture supernatant of HepAD38 cells induced for a minimum of 12 days in induction medium (DMEM–F-12 medium plus 4% Tet-free FBS). Virus was precipitated overnight in 10% polyethylene glycol 8000 (PEG 8000) at 4°C, concentrated by centrifugation, and finally dissolved in treatment medium (DMEM, 5% FBS). The titer of concentrated virus was determined by using the above-mentioned TaqMan qPCR method, and concentrated virus was used directly to infect cells or stored at −80°C.

HepG2-NCTP cells were infected with purified HepAD38 virus at a multiplicity of infection (MOI) of 50 in infection medium (DMEM, 5% FBS, 2% DMSO, 5% PEG 8000). Following infection, HepG2-NTCP cells were washed twice and switched to HepG2-NTCP treatment medium (DMEM, 5% FBS). PHHs were infected with HBV at an MOI of 50 in infection medium and grown in PHH maintenance medium for the duration of the experiment. Compounds were added after infection. Cells were retreated with compounds after 4 days. HBeAg and HBsAg in the supernatant at day 4 and day 8 postinfection were measured by enhanced chemiluminescence (ECL) enzyme-linked immunosorbent assays (ELISAs) (Autobio Diagnostics or Beijing Wantai Biological Pharmacy Enterprise Co.). Intracellular HBV DNA and pgRNA were measured by branch DNA (bDNA) assays (Thermo Fisher). Human serum protein shift assays were performed using human platelet lysate (HPL) (Cook Medical) as a source of human serum proteins. HepAD38 cells were seeded in induction medium containing 2% or 20% HPL (without FBS).

### HBV RNA/DNA analyses.

Total cellular RNA, cytoplasmic encapsidated HBV pgRNA, core DNA, and protein-free Hirt DNA were extracted as described previously (38, 39). Non-cccDNA in Hirt DNA was removed via T5 exonuclease (New England BioLabs) digestion. EcoRI endonuclease (New England BioLabs) was then used to linearize HBV cccDNA before electrophoresis.

For HBV RNA Northern blot analysis, total cellular RNA or encapsidated pgRNA samples were resolved in a 1.5% agarose gel containing 2.2 M formaldehyde and transferred onto a Hybond-XL membrane (GE Healthcare). For DNA Southern blot analysis, HBV core DNA or cccDNA samples were resolved by electrophoresis in a 1.2% agarose gel and blotted onto a Hybond-XL membrane. Membranes were probed with either an [α-^32^P]UTP (3,000 Ci/mmol; PerkinElmer)-labeled plus-strand-specific (for Northern blot hybridization) or minus-strand-specific (for Southern blot hybridization) HBV riboprobe and exposed to a phosphorimager screen. Extracellular HBV DNA and RNA were copurified from the culture medium of induced HepAD38 cells by using a MinElute virus vacuum kit (Qiagen). Viral DNA was quantified using the above-mentioned qPCR method directly, and HBV RNA was detected by RT-qPCR with the same core region primer/probe set after the removal of DNA contamination by DNase I (Thermo Fisher) treatment.

### HBV capsid gel assay.

The cytoplasmic HBV capsid particles were resolved in a native agarose gel by electrophoresis and transferred onto a nitrocellulose membrane, followed by a capsid enzyme immunoassay (EIA) using antibodies against core (Dako). For capsid-associated DNA detection, the membranes were treated with denaturing buffer (0.5 M NaOH, 1.5 M NaCl) and neutralization buffer (1 M Tris-HCl buffer [pH 7.4], 1.5 M NaCl), and the capsid-associated DNA was detected by the above-mentioned Southern blot analysis.

### Western blotting.

Whole-cell lysate samples prepared in Laemmli buffer were resolved in a 12% gradient SDS-PAGE gel, and proteins were transferred onto a polyvinylidene difluoride (PVDF) membrane (Bio-Rad). The membranes were blocked with Sea Block blocking buffer (Thermo Scientific) and probed with specific antibodies against HBV core amino acids 1 to 149 (Dako) or GAPDH (Thermo Scientific). Bound antibodies were revealed using IRDye secondary antibodies (Li-Cor). The immunoblot signals were visualized and quantified with the Li-Cor Odyssey system.

### Generation of HBV expression vectors and cell lines.

DNA constructs expressing HBV genotypes A, B, C, and D were designed based on sequences reported under GenBank accession numbers AB246337, AB246341, AB246345, and AB246347, respectively. Using gBlock gene fragments (IDT DNA), these sequences were cloned into a modified version of the LJ144 plasmid that supports HBV replication as well as the release of viral particles into the culture supernatant (40). This plasmid was also used to generate CI binding pocket site mutations. The DNA construct expressing NrtI^R^ HBV (rtL180M/M204V) was engineered by site-directed mutagenesis using the pTRE-HBV plasmid (41), which contained a Tet-responsive promoter and the HBV Gt-D genome (subtype ayw). Preseeded HepG2 cells were transfected with pTRE-HBV NrtI^R^ (rtL180M/M204V) using Lipofectamine 2000 (Invitrogen) and selected in medium containing 500 μg/ml G418 to generate a stable cell line expressing NrtI^R^ (HepG2-NrtI^R^). Colonies exhibiting high levels of HBV expression were further expanded.

### Transient HBV replication assay.

Huh7 cells were transfected using polyethylenimine (PEI) (4:1 reagent-to-DNA ratio; Polysciences) (42). Cells were treated with DMSO, ABI-H0731, other CIs, or ETV at 24 h posttransfection and retreated at 4 or 5 days posttransfection. Cells were harvested and lysed in 0.5% NP-40 lysis buffer (Dulbecco's phosphate-buffered saline, 0.5% [vol/vol] NP-40) at 7 or 8 days posttransfection, and the input plasmid DNA in the cell lysate was removed by DNase I (Sigma) digestion in the presence of 6 mM Mg(CH_3_COO)_2_ at 37°C for 60 min. HBV replicative DNA in the cell lysate was extracted using QuickExtract DNA extraction solution (Epicentre) and quantified using the above-mentioned TaqMan qPCR method.

### Combination assays.

Combination assays were performed using the HepAD38 cell line. The concentration ranges of ABI-H0731 and ETV were chosen based on previously determined EC_50_ values for each compound in this system: ABI-H0731 at 17 to 2,000 nM and ETV at 0.02 to 80 nM for MacSynergy II analysis and ABI-H0731 at 20 to 1,600 nM and ETV at 0.12 to 10 nM for CompuSyn analysis. Concentrations of working stocks for both drugs were adjusted so that the final DMSO concentration in HepAD38 cells remained at 1%. The CompuSyn analysis was performed using data points at which the ratios of both drugs remained constant.

### Incoming capsid assay.

HepG2-NTCP or PHH cells were infected with HBV (Gt-D) from induced HepAD38 cells at an MOI of 2,500 and then treated with compounds at 1 h postinfection. The cells were then harvested and lysed with lysis buffer (10 mM Tris-HCl [pH 8.0], 1 mM EDTA [pH 8.0], 1% NP-40, 50 mM NaCl) at the indicated time points. The cell lysate was subjected to native agarose electrophoresis, and the capsid-associated HBV DNA was detected by the above-mentioned Southern blot analysis.

### cccDNA formation assay in infected HepG2-NTCP and PHH cells.

HepG2-NTCP cells or PHH cells were infected with HBV at MOIs of 500 and 1,000, respectively, and then treated with compounds at 3 h postinfection. Infected cells were harvested 4 days later, and cccDNA was prepared and detected by the above-mentioned Southern blot assay.

### CYP450 inhibition and induction assays.

A series of studies was conducted to determine the CYP450 drug-drug interaction potential of ABI-H0731 in human liver microsomes. Standard methods (27) were used to investigate the inhibition potential of ABI-H0731 up to 100 μM.

The determination of CYP induction potential was conducted using CPX2 cells that harbor the human PXR gene and a luciferase reporter gene linked to two promoters identified in the human 3A4 gene. Concentrations of ABI-H0731 of up to 40 μM were incubated for 24 h.

### CYP450 microsomal and hepatocyte assays.

ABI-H0731 (dissolved in DMSO with a final solvent concentration in the incubation mixture of <0.1% [vol/vol]) was assessed for metabolic stability in liver microsomes from mouse, rat, dog, cynomolgus monkey, and human. *In vitro* intrinsic clearance (CL_int_) was determined as follows. Briefly, incubation mixtures contained liver microsomes (0.5 mg protein/ml) and ABI-H0731 (0.5 μM), with aliquots sampled at 0, 15, 20, 40, and 60 min. The calculated CL_int_ was determined from the relationship CL_int_ = *k* × (volume of incubation in ml/mg microsomal protein) × (mg protein in liver/g liver weight) × (g liver weight/kg body weight). The values for liver weights, body weights, as well as hepatic blood flows (*Q*_H_) across species were obtained from data reported previously by Davies and Morris (28). Values for liver microsome protein/liver weight for mouse, rat, dog, and monkey were 45 mg/ml (27), and that for human was 33 mg/g. The calculation used for the prediction of hepatic clearance (CL_pred_) is CL_pred_ = (*Q*_H_ × CL_int_)/(*Q*_H_ + CL_int_). Hepatocyte incubation mixtures were sampled at 15, 30, 60, and 120 min, and incubation mixtures contained 1 million cells/ml and 1 μM ABI-H0731.

### Plasma protein binding.

The fraction of ABI-H0731 unbound in plasma was determined in mouse, rat, cynomolgus monkey, and human plasma (K_2_EDTA) at concentrations of 2, 10, and 30 μM using a rapid equilibrium dialysis (RED) device (Thermo Scientific, Rockford, IL). Plasma and ABI-H0731 were incubated at 37°C on an orbital shaker for 6 h.

### Pharmacokinetics.

All animal procedures were conducted under institutional approved IACUC protocols.

ABI-H0731 was formulated in *N*-methylpyrrolidone (NMP)–Solutol HS-15–normal saline (5:5:90) between the ranges of *X* and *Y* mg/ml and dosed between 0.5 and 2 mg/kg of body weight for intravenous administration and between 2 and 5 mg/kg for oral gavage. Pharmacokinetic studies were performed at Wuxi (Shanghai, China) for studies in male mouse, rat, and cynomolgus monkey and at Sai (Pune, India) for studies in male dog. For all oral studies, the formulation was administered in the fasted state (food removed at least 12 h prior to dosing) to groups of 3 per arm.

## Supplementary Material

Supplemental file 1
