# Supplementary material for: Preclinical Profile and Characterization of the Hepatitis B Virus Core Protein Inhibitor ABI-H0731
Source: Antimicrob Agents Chemother. 2020 Oct 20;64(11):e01463-20. doi: 10.1128/AAC.01463-20 (PMC7577125; doi:10.1128/AAC.01463-20)
Supplement: Supplemental file 1 [file AAC.01463-20-s0001.pdf]

Supplemental Material

Title: Preclinical Profile and Characterization of the HBV Core Protein Inhibitor ABI-H0731

Running title: Preclinical Profile of ABI-H0731

Authors: Qi Huang, Dawei Cai, Ran Yan, Lichun Li, Yuhua Zong, Lida Guo, Alexandre Mercier*, Yi Zhou*, Ariel Tang, Kirk Henne* and Richard Colonno[#]

[#] Corresponding Author

Affiliations: Current or former employees of Assembly Biosciences, South San Francisco, CA

KEYWORDS: Core Protein (Cp), Core inhibitor, cccDNA, hepatitis B virus, pregenomic RNA (pgRNA)

Supplementary Tables

Table S1. GLS4, NVR 3-778 and ETV potency in HBV expression/infection models

Marker	EC_{50} (nM) ± SD								
	GLS4			NVR 3-778			ETV		
	HepAD38	HepG2-NTCP	PHH	HepAD38	HepG2-NTCP	PHH	HepAD38	HepG2-NTCP	PHH
**Viral DNA**	29 ± 9	58 ± 6	1,940 ± 40	238 ± 33	590 ± 71	1,683 ± 113	1.2 ± 0.3	0.8 ± 0.2	<0.1
**HBeAg**	n/a	549 ± 48	1,480 ± 170	n/a	8,364 ± 308	4,010 ± 7	n/a	>>100	>>100*
**HBsAg**	n/a	673 ± 41	1,860 ± 430	n/a	9,258 ± 1050	5,040 ± 1474	n/a	>>100	>>100*
**pgRNA**	n/a	279 ± 43	1,930 ± 30	n/a	7,605 ± 771	4,204 ± 101	n/a	>>100	>>100*

n/a = not applicable
*incomplete inhibition

Table S2. Effect of Serum Proteins on ABI-H0731 Activity in HepAD38 Cells

EC_{50} (nM) ± SD		Fold Shift
2% HPL	20% HPL	
200 ± 5	522 ± 40	2.6

Supplementary Figures

Figure S1

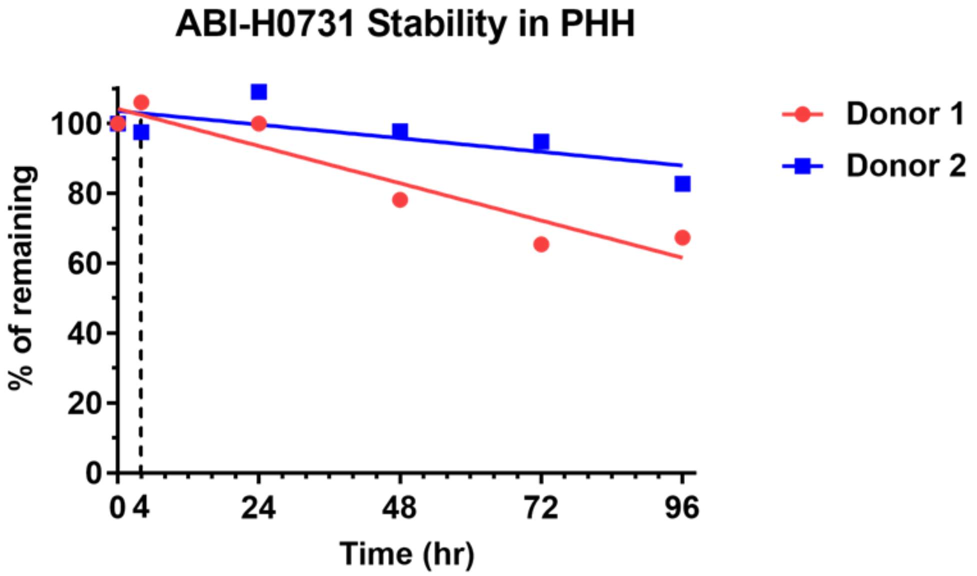

Figure S1 Legend: ABI-H0731 stability in Human Primary Hepatocyte (PHH). . PHHs which derived from two different donors were incubated with 10 µM ABI-H0731 and supernatant was collected at 4, 24, 48, 72 or 96 hours post treatment. The remaining ABI-H0731 was measured by Mass Spectrometry.

Figure S2

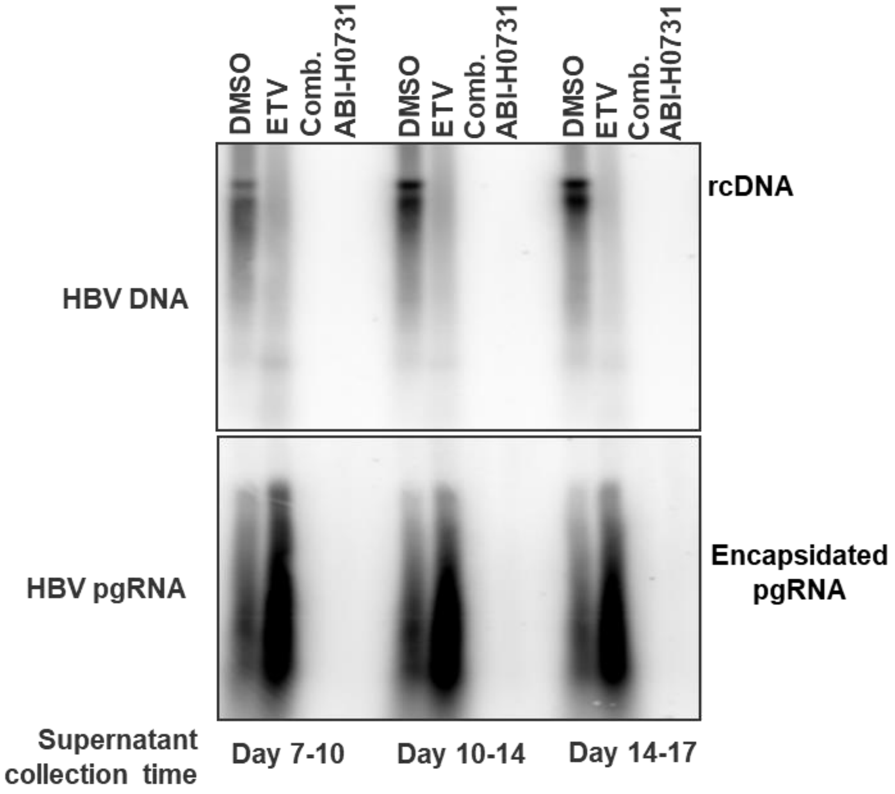

Figure S2 Legend: HepAD38 cells were induced by removal of tet and treated with ETV (100 nM), ABI-H0731 (10 µM) or combination simultaneously. The treatment was repeated every 3 to 4 days and the supernatant was collect at the same time. Viral DNA and RNA were co-purified from the cell supernatant collected on day 10, day 14 and day 17 and viral DNA was analyzed by Southern analysis directly (top panel). After removal of viral DNA by dsDNase, viral encapsidated RNA was detected by Northern blot analysis (bottom panel). rcDNA: relaxed circular DNA.

Figure S3

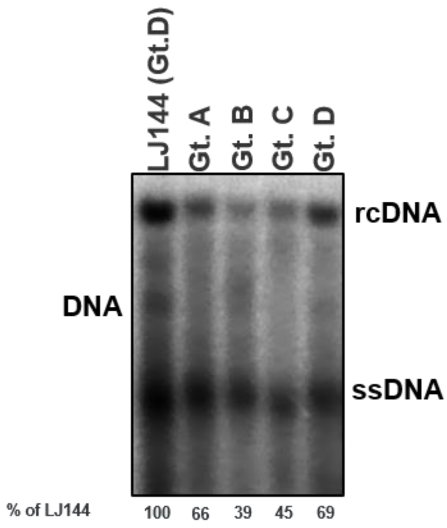

Figure S3 Legend: Replicative fitness of HBV genotype A, B, C and D stains. Lab strain HBV expression vector (LJ144, genotype D, serotype *ayw*) and its derived genotype A, B, C and D stain expression constructs were transfected into HepG2 cells, intracellular viral core DNA were extracted 6 days post transfection and detected by Southern blot analysis. Band density was quantified by ImageJ software. rcDNA: relaxed circular DNA; ssDNA: single-stranded DNA.

Figure 4S

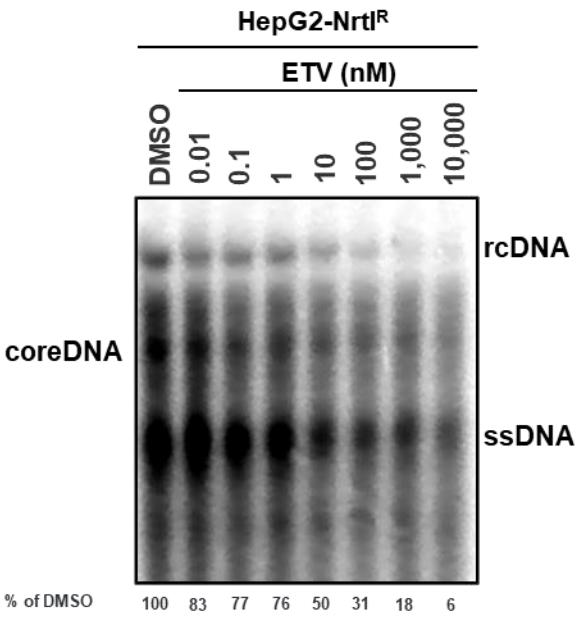

Figure S4 Legend: Characterization of HepG2-NrtI^R cell line. Tetracycline inducible HepG2-NrtI^R cell line which supports NrtI^R HBV (rtL180M/M204V) replication was generated as described in material and methods section. HepG2-NrtI^R cells were induced by removal of tet and treated with the indicated concentration of ETV or left as DMSO control simultaneously. Intracellular viral core DNA were extracted 7 days post induction and detected by southern blot analysis. Band density was quantified by ImageJ software. The EC_{50} of ETV against this NrtI^R HBV is about 10 nM, which is 10 x higher than ETV against a wild-type strain (EC_{50} = 1nM). rcDNA: relaxed circular DNA; ssDNA: single-stranded DNA.
